# Content of Bioactive Compounds in Highbush Blueberry *Vaccinium corymbosum* L. Leaves as a Potential Raw Material for Food Technology or Pharmaceutical Industry

**DOI:** 10.3390/foods13020246

**Published:** 2024-01-12

**Authors:** Maria Czernicka, Patrycja Sowa-Borowiec, Czesław Puchalski, Zbigniew W. Czerniakowski

**Affiliations:** 1 Department of Bioenergetics, Food Analysis and Microbiology, University of Rzeszow, 35-601 Rzeszow, Poland; cpuchal@ur.edu.pl; 2 Department of General and Inorganic Chemistry, Faculty of Chemical Engineering and Technology, Cracow University of Technology, 31-155 Cracow, Poland; patrycja.sowa-borowiec@pk.edu.pl; 3 Department of Agroecology and Forest Utilization, University of Rzeszow, 35-601 Rzeszow, Poland

**Keywords:** *V. corymbosum* L., blueberry leaves, bioactive compounds, arbutin, hydroquinone

## Abstract

This study was performed to investigate the content of selected phenolic compounds, antioxidant activity and the levels of arbutin and hydroquinone in 25 varieties of highbush blueberry (*Vaccinium corymbosum*) leaf samples. An analysis of the bioactive components was performed using the HPLC technique and the antioxidant activity was determined via spectrophotometric methods. The content of chlorogenic acid in the analysed leaf extracts ranged from 52.76 mg/g (Spartan variety) to 32.37 mg/g (Nelson variety) and was present in the highest concentration among all the analysed phenolic acids. Particularly large levels of isoquercetin were found in the Aurora, Ivanhoe and Toro varieties (28.40 mg/g, 26.24 mg/g and 21.57 mg/g, respectively). An exceptionally high rutin content (*p* < 0.05) was found in the Ivanhoe variety (27.19 mg/g) as compared to the other varieties, where it ranged from 2.06 mg/g (Earliblue and Patriot varieties) to 10.55 mg/g (Bluejay variety). The Patriot variety was determined to possess the highest antioxidative activity using the FRAP method (1086.15 μmol Trolox/g d.w.) and based on its DPPH radical scavenging activity (1124.17 μmol Trolox/g d.w.). The total phenolic content (TPC) determined via spectrophotometry ranged from 48.11 mg GAE/g d.w. (Elizabeth variety) to 177.31 GAE/g d.w. (Patriot variety). The arbutin content in the leaves of all tested varieties exceeded 2%, so it can be concluded that they constitute a stable source of arbutin. Three varieties (Bonus, Chanticleer and Herbert) can be considered a potential alternative to bearberry and lingonberry leaves. The hydroquinone content in the analysed extracts was determined to be at a lower level. *V. corymbosum* leaves can be considered an interesting herbal material for use in traditional herbal medicinal products but not directly for food products and dietary supplements.

## 1. Introduction

The highbush blueberry was once a regional crop in the United States, but is now a specialty crop grown around the world. The expansion of the crop’s range is driven by increasing demand for blueberries worldwide, the availability of versatile varieties and the adoption of new, intensive production systems. Southern highbush blueberry cultivation has expanded into non-traditional growing areas worldwide due to premium cultivars and improved horticultural practices [1]. Consumption of blueberries has increased rapidly worldwide over the last eight years. North America is the traditional market, where about 58% of all fresh blueberries are consumed, even as demand has grown rapidly in new markets such as Europe and China. The growing demand is accompanied by increased production in both traditional and new growing areas around the world [2]. Highbush blueberries have played a key role in this expansion due to the high quality of their fruit for fresh markets, their adaptation to growing in both hot and temperate climates, and due to their scientifically proven health-promoting properties. The health benefits of these fruits are attributed to antioxidants, which mitigate the undesirable effects of reactive oxygen species by highly effectively controlling free radical production and supporting the antioxidant and detoxifying mechanisms in the organism [3,4,5]. In turn, phenolic compounds of plant origin and their antioxidant properties are an intensely researched topic, with increasing evidence supporting their protective effects against many non-communicable diseases in humans [6,7,8]. These fruits are used for therapeutic purposes due to the high content of phenolic compounds, many of which can be found in a significant body of scientific evidence regarding the health benefits of Vaccinium species related to their consumption [9,10], while a smaller body of evidence highlights the proficiency of bioactive compounds found in the leaves [11]. While the *Vaccinium* fruits have a seasonal nature, which implies high harvesting and storage costs, the leaves are available in most seasons and some even in wintertime (e.g., lingonberry and bearberry). According to Stefanescu et al. [11], who reviewed research on Ericaceae species’ leaves, their chemical composition revealed three main phenolic compounds—chlorogenic acid, quercetin and arbutin—and their health-promoting functions, such as antioxidant and anti-inflammatory effects, especially on neurodegenerative diseases, cancer and obesity, were confirmed by in vitro and in vivo studies [12,13,14]. Among numerous plants of the *Ericaceae* family, bearberry (*Arctostaphylos uva-ursi* (L.) Spreng) and lingonberry (*Vaccinium vitis-idaea* L.) leaves are of particular importance due to their medicinal properties [15,16,17]. An issue in harvesting herbal materials from these plants is that the former is a protected plant in numerous countries, while the range of the latter is limited. Consequently, our attention was drawn to the highbush blueberry (*V. corymbosum* L.), which is a plant grown for consumption in many regions of the world. Highbush blueberry fruits enjoy fairly high demand due to their flavour and health-promoting properties, as previously described. Their leaves, on the other hand, rarely attract interest, even though they can be easily obtained during bush screening and seedling production. It is also known that leaves appear on blueberry bushes for a longer period and outweigh the amount of fruit, so their availability prompts the search for alternative uses for this type of raw material. It was also found that the highest fruit setting ratio of 4.8 ± 1.0 was observed when the leaf-to-fruit ratio was 6.2 ± 0.9 [18], which shows the need to care for the quality and quantity of leaves on bushes in blueberry cultivation.

Fruit bush leaves from horticultural production can find an alternative use as material for pharmaceutical purposes, although they need to meet strict pharmaceutical requirements [19]. In food production, the requirements imposed on materials can be slightly less strict, especially when the plants are intended for producing plant-based concentrates of bioactive compounds used for food enrichment. Furthermore, as shown in other studies, it is also possible to selectively extract bioactive components without adversely affecting the health-promoting compounds, which makes it possible to effectively eliminate their presence in biocomponent-enriched products [20,21].

The leaves of orchard and forest plants already find use in nutrition as components of leaf infusions, which are a healthy alternative to teas and are made by fermenting the leaves of selected fruit bushes and/or medicinal plants. Through a process of wilting, rolling and drying, they not only obtain a higher concentration of ingredients beneficial to health, such as tannins, but also health-promoting ingredients, for example, numerous phenolic compounds and glycosides. It needs to be added that food production is more liberal in using plant additives with pro-health potential, often without considering the potential presence of harmful or toxic substances, such as plant hormones, saponins or alkaloids [22,23]. It is also known that other food ingredients and treatment processes applied to foods containing pro-health additives, as well as food preservation processes, can effectively limit the bioavailability of these pro-health or even harmful substances, but they should never be ruled out or ignored [24,25].

The sparse papers on biologically active compounds in highbush blueberry leaves indicate that the leaves contain chlorogenic acid and quercetin and its glycosides (rutin, hyperoside and isoquercetin) [26]. *V. corymbosum* leaf extracts above all exhibit significant antibacterial and antioxidative activity [27]. The antioxidative activity of the compounds found in this plant’s leaves have found use even in the production of functional packaging material [28].

These discoveries demonstrate that *V. corymbosum* leaf extracts can have a very broad range of applications in medicine and the food industry. However, the potentially high arbutin content in the leaves, and consequently hydroquinone as well, a compound suspected of carcinogenicity [29], can raise justified concerns. Considering the beneficial properties of substances found in the leaves of highbush blueberries and the importance of substances with potentially toxic effects, in our experiment, we attempted to analyse these issues in a wide spectrum of varieties of *V. corymbosum* L. that are popular and widely distributed among growers around the world, not all of which have been thoroughly researched to date. In our work, we attempted to characterise the antioxidant capacity, i.e., the ability to neutralise a synthetic radical, and the health-promoting properties and toxic substances that also occur in highbush blueberry leaves. The aim of this study was therefore to investigate the qualitative and quantitative content of pro-health compounds in *V. corymbosum* leaves and to determine their levels of arbutin and hydroquinone.

## 2. Materials and Methods

### 2.1. Plant Samples

The study material comprised samples of 25 varieties of highbush blueberry leaves from a plantation located in a mountainous area within the Carpathian Foothills region in southeastern Poland, from clean areas with low industrial development (GPS: 50°06′38″ N 22°10′53″ E). The Carpathian Foothills are the lowest portion of the Polish Carpathians, forming a group of low-altitude hills between 350 and 600 m a.s.l., with smooth and round slopes. The Carpathian Foothills are characterised by moderate, intermediate and submontane climates. The leaves were taken from highbush blueberry (*V. corymbosum*) bushes after the fruit harvest in August–September 2021 so that the defoliation would have no negative effect on the fruit crop. Twenty plants of each variety were sampled, from which 200 leaves of similar size (10 per plant) were obtained for drying. The harvesting was conducted in triplicate over three consecutive weeks. The varieties and harvest times are shown in Table 1.

The plant material was preserved by drying it in a DanLAB (Poland) air circulation laboratory dryer at 35 °C. Next, the water content in the dried material was determined, which served as an indicator for the end of the drying process, as the water content did not exceed 8%. Having considered the results of experiments involving test material available commercially in dried form [30] and the significant heterogeneity of the dried material, the water content range of 6–8% was deemed sufficient for preserving the test material and concluding the drying process. Portions of dried test material of each variety were fragmented in an IKA type A 11 Basic Analytical Mill (Königswinter, Germany), then sieved through a 0.18 mm sieve. For extraction, 2 g portions of homogeneous dried plant material were moved to extraction tubes and covered with 20 mL of deionised water, sealed and placed for 60 min in a Polsonic Sonic 22 (Warsaw, Poland) ultrasound bath with the thermostat function set at 40 °C. Extraction was performed using ethanol with a 50% alcohol content. After completion of the ultrasound-assisted extraction, the samples were transferred to a Biosan ES-20/60 rotary shaker (Riga, Latvia) and mixed under similar conditions as before for 30 min at 40 °C at 180 revolutions per 1 min. After the extraction was concluded, the samples were purified under pressure on filter papers placed on a Buchner filter, ensuring that the extraction material was thoroughly dried from the solvent, and then the filtrates were centrifuged in an Eppendorf 5702 laboratory centrifuge (Hamburg, Germany) for 10 min, RCF = 2600 g. After centrifuging, the supernatant was poured into separate, clean tubes. The clear highbush blueberry extracts were applied in their entirety to conditioned Waters Sep-Pak (C18, 500 mg) syringe filter cartridges (Milford, MA, USA). Phenolic compounds were leached from the columns with methanol directly in a round-bottomed flask and were concentrated at 40 °C until the solvent was evaporated in a Hei-VAP Precision rotational vacuum evaporator from Heidolph (Schwabach, Germany). The leaf extracts in the round-bottomed flasks were dissolved with 50% hydrated acetonitrile and filtered through PTFE socket filters with a 0.45 µm pore size directly prior to chromatographic analysis.

### 2.2. Analytical Procedures

#### 2.2.1. Profiles of Phenolic Compounds

The contents of phenolic compounds were determined according to the method described by Piechowiak et al. [31], with some of our own modifications. Selected polyphenolic compounds were determined in the highbush blueberry extracts via high-performance liquid chromatography (HPLC) using a SYKAM S 600 analyser (Sykam GmbH, Eresing, Germany) equipped with a photodiode detector (S 3210) operating within the 190 to 900 nm wavelength range, a pump (S 1132) and a column thermostat (S 4120). Before the analysis, the samples were filtered through a nylon syringe filter with a 0.22 µm pore diameter. The bioactive compounds were separated in a Bionacom Velocity STR C18 column (3.0 × 100 mm; 2.5 μm) with an injection volume of 20 µm, flow rate of 0.5 mL/min. and column thermostat temperature of 25 °C. Mobile phase composition was water acidified with formic acid (0.1% *v*/*v*) (A) and acetonitrile (B). The separation was performed using the following gradient: 10% B (1.5 min); 10–30% (1.5–9 min); 30–50% (9–20 min); 50–10% (20–25 min). Quantitative determinations were carried out using the calibration curves of individual standards ranging from 0.005 to 0.1 g·L^−1^ (R^2^ ≥ 0.9998).

#### 2.2.2. Determination of Arbutin and Hydroquinone Content

Arbutin and hydroquinone determinations were performed based on the methodology described in the latest 7th edition of the European Pharmacopoeia [32]. A 0.800 g weighed amount of powdered plant material was placed in a 100 mL round-bottomed flask, 20 mL distilled water was added, and the flask was heated for 30 min in a water bath under a reflux condenser. After cooling, the extract was filtered through a wad of cotton wool and was extracted again, together with the residue in the flask, with another 20 mL of distilled water for 30 min in a water bath under a reflux condenser. After cooling, all the liquid was filtered through a paper filter and left to cool, then topped up with 50 mL of distilled water and filtered again, discarding the first 10 mL of the filtrate. The purified plant extracts were applied to conditioned Waters Sep-Pak (C18, 500 mg) syringe filter cartridges (Ireland). Phenolic compounds were leached from the columns with methanol in 10 mL round-bottomed flasks and were concentrated at 40 °C until the solvent was evaporated in a Hei-VAP Precision rotational vacuum evaporator from Heidolph (Schwabach, Germany). The leaf extracts in the round-bottomed flasks were dissolved with methanol and filtered through PTFE socket filters with a 0.45 µm pore size directly prior to chromatographic analysis. A standard solution was made by dissolving 50 mg of arbutin in 50 mL of mobile phase and 2.5 mg of hydroquinone in 10 mL of mobile phase, then mixed at the ratios specified in the methodology. The plant extracts were separated using a Thermo Scientific/Dionex UltiMate 3000 liquid chromatograph with a UV detector and a DAD-3000 (RS) diode matrix (ESA, Chelmsford, MA, USA). A HALO 90 A C18, 2.7 μm, 4.6 × 150 mm column was used for the separations at 25 °C. The mobile phase was methanol and water (10:90 *v*/*w*), the flow rate was 0.8 mL/min, and the assay time was 30 min, with detection at a wavelength of 280 nm. The injection volume was 10 µL. The average recovery for the highbush blueberry was 97%. The operation of the chromatographic set and processing of the obtained data were coordinated using the Thermo Scientific Dionex Chromeleon 7.2 Chromatography Data System. 

#### 2.2.3. Determination of Antioxidant Activity

The antioxidative activity was determined using FRAP (Ferring Ion Reducing Antioxidant Power), while the DPPH (2,2-diphenyl-1-picrylhydrazyl) radical scavenging activity was determined in accordance with the methodology described by Sowa et al. [33]. The antioxidative activity results were expressed as Trolox equivalents (TE) per 1 g of dry weight of the plant material (μmol TE/g d.w.) based on a Trolox solution calibration curve within a 25–300 nmol/mL (FRAP) and 5–60 nmol/mL (DPPH) range. The measurements were performed using a UV6000 UV VIS spectrophotometer (Shanghai Metash Instruments Co., Ltd., Shanghai, China).

#### 2.2.4. Analysis of Total Phenolic Compounds (TPC)

The content of total phenolic compounds (TPC) was investigated using Folin–Ciocalteu’s method, as described by Stratil et al. [34]. The results were expressed as 1 mg of gallic acid equivalents per 1 g of dry weight of weight of the plant material (mg GAE/g d.w.) of tested samples, using a calibration curve plotted for GAE solution in a concentration range of 25–250 µg/mL. The measurements were performed using a UV6000 UV VIS spectrophotometer (Shanghai Metash Instruments Co., Ltd., Shanghai, China).

### 2.3. Chemicals and Reagents

Analytical-purity reagents (analytical standards) designed for liquid chromatography were used for the determinations: hydrochloric acid, formic acid, ethanol and acetonitrile from Sigma-Aldrich (Poznan, Poland) and methanol from J.T. Baker (Phillipsburg, NJ, USA). 6-hydroxy-2,5,7,8-tetramethylchroman-2-carboxylic acid (Trolox), 2,4,6-tri(2-pyridyl)-s-triazine (TPTZ) and 2,2-diphenyl-1-picrylhydrazyl (DPPH) were purchased from Sigma-Aldrich (Poznan, Poland). Analytical standards of chlorogenic acid, neochlorogenic acid, cryptochlorogenic acid, 3,5-dicaffeoylquinic acid, 4,5-dicaffeoylquinic acid, catechin, rutin and isoquercetin for HPLC were obtained from Extrasynthese (Genay, France). Arbutin standard was obtained from CPAchem Ltd. (Bogomilovo, Bulgaria) and hydroquinone standard from Sigma-Aldrich (Poznan, Poland). Deionised water obtained from a Hydrolab Polska HLP 5P (Hydrolab, Poznan, Poland) deioniser was used.

### 2.4. Statistical Analysis

All analyses were performed in three independent replications for each sample. The contents of phenolic compounds, arbutin hydroquinone content and antioxidant activity were expressed as the mean ± standard deviation. The acquired findings were subjected to statistical analyses with the use of Statistica v13.1 (StatSoft Inc., Tulsa, OK, USA). Significant differences between types of highbush blueberry leaves based on the tested parameters were obtained through a one-way analysis of variance (ANOVA), followed by the Tukey multiple comparison test. The multivariate statistical analysis was performed using cluster analysis (CA), combined with a heat map with the use of Statistica v13.1 (StatSoft Inc., Tulsa, OK, USA) and principal component analysis (PCA) with the use of OriginPro 2023 (OriginLab Corporation, Northampton, MA, USA). Correlations between tested parameters were established using Pearson’s correlation test and presented as a triangle heat map with the use of OriginPro 2023 (OriginLab Corporation, Northampton, MA, USA).

## 3. Results and Discussion

In the analysed highbush blueberry Vaccinium corymbosum L. leaf extracts, compounds belonging to phenolic acids were identified, mainly chlorogenic acid derivatives and flavonoids, primarily quercetin glycosides. The content of individual phenolic compounds varied depending on individual variety and, in most cases, statistically significantly differed between varieties (*p* < 0.05) (Table 2 and Table 3). The results of our analyses confirm the information reported by Ferlemi et al. [26] that chlorogenic acid, rutin and isoquercetin are found in *V. corymbosum* leaves. Therapeutic properties of rutin, also known as rutoside, have been proven for a number of pro-health effects, such as prevention of neuroinflammation and anticancer, antidiabetic, antimicrobial, organ protection and nutraceutical effects [35]. In turn, isoquercetin has shown broad-spectrum antiviral activity against infection by influenza, Zika and Ebola. It is suggested that administration of these flavonols could prevent infection by severe acute respiratory syndrome-coronavirus-2 (SARS-CoV-2) or arrest the progression to severity and lethality of the resulting coronavirus disease [36]. Chlorogenic acid is the most abundant compound belonging to phenolic acids found in the plant material, and has a very broad range of pharmacological applications based on antioxidant, liver and kidney protection; antibacterial and antitumour effects; regulation of glucose metabolism and lipid metabolism; an anti-inflammatory effect; protection of the nervous system; and action on blood vessels [37,38,39,40,41]. Its content in the analysed leaf extracts ranged from 52.76 mg/g (Spartan variety) to 32.37 mg/g (Nelson variety). This acid was present in the highest concentration among all the analysed phenolic acids, which is consistent with results produced by Wang et al. [42], who analysed 104 blueberry varieties. In the leaves tested for this report, it was accompanied by cryptochlorogenic acid and neochlorogenic acid, which usually form a complex of phenolic acids in many traditional plant-based medicines according to Jie et al. [43].

The neochlorogenic acid content varied greatly. The highest content of this acid was found in the Bonus variety, which is classified as an early cultivar (9.03 mg/g), while in the Hannah’s Choice variety, this acid was <LOQ. The cryptochlorogenic acid content also differed between varieties. The lowest levels were found in the Elizabeth and Nelson varieties (1.99 mg/g), while the highest content was identified in Darrow and Hannah’s Choice (4.31 mg/g and 4.30 mg/g, respectively). Additionally, we demonstrated the presence of 3,5-dicaffeoylquinic acid and 4,5-dicaffeoylquinic acid in the material, which are chlorogenic acid isomers of low stability, but which also exhibit a broad range of therapeutic effects [44]. Significant variation was observed for these compounds as well, especially in the case of 4,5-dicaffeoylquinic acid, which was present at fairly high levels (above 7 mg/g) in the Bluejay and Chandler varieties, while in some others (Bluegold, Draper and Hannah’s Choice), it was below LOQ.

A similarly broad range of potentially beneficial health effects is found in the flavonoids isoquercetin and rutin [45,46,47], with isoquercetin found in noticeably greater quantities in the tested materials. Particularly large levels of this substance were found in the Aurora, Ivanhoe and Toro varieties (28.40 mg/g, 26.24 mg/g and 21.57 mg/g, respectively). Nelson and Denis Blue contained the lowest quantities (5.13 mg/g and 8.75 mg/g, respectively). An exceptionally high rutin content (*p* < 0.05) was found in the Ivanhoe variety (27.19 mg/g) as compared to the other varieties, where it ranged from 2.06 mg/g (Earliblue and Patriot varieties) to 10.55 mg/g (Bluejay variety). Among the flavonoids identified, catechin was present at the lowest levels, although, as with the other compounds, a marked variation between the test varieties was observed (*p* < 0.05 statistically significant differences in most cases). The highest catechin content was found in the Hannah’s Choice variety (4.15 mg/g), while the lowest was observed in the Duke variety (1.35 mg/g).

In general, a higher content of phenolic acids than flavonoids was observed in the analysed varieties. A similar situation was noted in the study by Wu et al. [48]. However, the phenolic compound profile and the ratios of individual compounds they found were different than in the extracts we analysed (for example, it was found that neochlorogenic acid is more abundant in the leaf extracts). This is most likely a result of different extract preparation procedures, but it could also be affected by the time of harvesting or geographical area [11]. Furthermore, as Riihinen et al. [10] observed in their study, the factor deciding the phytochemical composition of the blueberry is the leaf tissue maturity. This study determined that tested leaves of V. corymbosum have increased flavonol and hydroxycinnamic acid (quercetin, kaempferol, p-coumaric, caffeic and ferulic) levels, compared to green leaves. In our study, we used a 50% *v*/*v* water–ethanol solution, which was dictated by the fact that water–ethanol solutions are very good at extracting bioactive compounds, and ethanol is used for preparing tinctures, as well as ethanol extracts and syrups in pharmacy. The study by Wu et al. [48] used an 85.00% methanol solution acidified with 0.1% formic acid. Similarly, in the study by Stefănescu et al. [11], slightly different profiles for the analysed compounds were produced for the six tested commercial blueberry (*Vaccinium corymbosum* L.) varieties. For example, chlorogenic acid was determined at much lower levels (between 0.44 and 1.23 mg/g), while rutin was found to be more abundant (14.44–35.77 mg/g). However, as in our study, they observed significant differences between varieties. Varying content levels of individual compounds in the leaves and fruits of *Vaccinium corymbosum* L., depending on the extracting agent used, were shown in the study by Tenuta et al. [49].

The arbutin and hydroquinone content determined in our study is shown in Table 4. The European Pharmacopoeia states that dried bearberry leaf, as a herbal material, should contain less than 7% (European Pharmacopoeia 7.8) [50], while the Polish Pharmacopoeia specifies a corresponding value of 4% arbutin for lingonberry [51]. The arbutin content in the leaves of all tested varieties exceeded 2%, so it can be concluded that they constitute a stable source of arbutin. Consumption of plant products containing arbutin results in a rapid increase in hydroquinone levels in the blood plasma [52,53]. Toxicity studies of hydroquinone administered to animals over prolonged periods of time demonstrated that it is a genotoxic compound with carcinogenic potential, although there is no basis for determining its permissible concentration in biological material [54]. For this reason, *V. corymbosum* leaves can be considered an interesting herbal material for use in traditional herbal medicinal products utilised for the relief of symptoms of mild recurrent lower urinary tract infections [55,56]. Three varieties (Bonus, Chanticleer and Herbert) can be considered a potential alternative to bearberry and lingonberry leaves. Leaf harvest could therefore be an additional source of income for highbush blueberry growers. The content of this compound, calculated to dry plant weight, ranged from 19.94 mg/g in the Denis Blue variety, classified as a late variety, to 44.24 mg/g in the Bonus variety, which belongs to the early varieties. However, no effect of the growing season duration was observed on the content of this compound. Studies concerning the arbutin content in *Vaccinium corymbosum* L. are scarce [57,58].

The hydroquinone content in the analysed extracts was determined to be at a lower level, with the lowest found in the Bluegold, Darrow, Herbert, Liberty and Spartan varieties (an average of 0.26 mg/g), while the highest was noted for the Chandler variety (0.88 mg/g) (*p* < 0.05). It is known that hydroquinone induces the formation of reactive forms of oxygen and quinones, which leads to oxidative damage to membrane lipids and proteins such as tyrosinase, while at the same time it inhibits the pigmentation process. Due to the risk of adverse effects, hydroquinone has been prohibited by the European Commission [59]. The majority of the scientific papers available analysed extracts from *Vaccinium corymbosum* L. leaves in terms of bioactive compounds and their pro-health activity (e.g., antioxidative activity, antimicrobial, anti-inflammatory, antidiabetic properties), and based on these results, gave recommendations for using leaf extracts from this species to supplement everyday diet, completely ignoring the content of a compound that can potentially be toxic, and products containing this compound should be used with care [11,27,48,60]. Hydroquinone content was confirmed in the study by Yavorska et al. [58], although no specific concentration in the test extracts was given. A study conducted by de Arriba et al. [61] assessed the potential toxicity of herbal preparations made from *Arctostaphylos Uva-ursi folium* (bearberry leaf), which contain high levels of arbutin, and at the same time, a small concentration of free hydroquinone (<0.3%).

The antioxidative activity, determined by two methods (FRAP and DPPH), of the analysed leaf extracts differed between varieties (Table 5), similarly to the preceding analyses. Most varieties differed statistically significantly (*p* < 0.05). The highest antioxidative activity was determined in the Patriot variety (1086.15 μmol Trolox/g d.w.) using the FRAP method, while the lowest was noted for the Elizabeth variety (350.77 15 μmol Trolox/g d.w.). For antioxidative activity determined against the DPPH radical, the highest activity was observed for the Patriot (1124.17 μmol Trolox/g d.w.) and Huron varieties (1144.47 μmol Trolox/g d.w.), while the lowest was noted for Denis Blue (400.74 μmol Trolox/g d.w.) and Elizabeth (403.65 μmol Trolox/g d.w.). This antioxidative activity largely resulted from the presence of polyphenolic compounds. The total phenolic content (TPC) determined via spectrophotometry ranged from 48.11 mg GAE/g d.w. (Elizabeth variety) to 177.31 GAE/g d.w. (Patriot variety) (Table 5). A significant correlation was observed between the antioxidative activity measurement results and the general phenolic compound content for the analysed leaf extracts. In a study by Wu et al. [48], the antioxidative activity determined by FRAP was consistent with our results, ranging from 358.2 to 1600 μmol FEAC/g d.w., while lower values were noted for DPPH, from 182.3 to 357.9 μmol TEAC/g d.w., although the antioxidative activity differed between varieties. The TPC value they determined was within the 32.18–185.2 mg GAE/g d.w. range. In the study by Stefănescu et al. [11], the ability to inhibit DPPH radicals was determined as a percentage of free radical sweeping. High values were found for three varieties: Toro, Elliott and Nelson, with 70.41%, 68.42%, and 58.69%, respectively. The highest TPC was determined for the Nelson and Toro varieties (13.555 mg GAE/100 g leaf material and 13.92 mg GAE/100 g leaf material, respectively). A study by Routray and Orsat [62] analysed the impact of the harvest time on the phenolic compound content for two varieties: Nelson and Elliott. They found that the phenolic content began to rise in September and October, reaching 152.356 mg GAE/g d.w. (Nelson variety) and 155.830 mg GAE/g d.w. (Elliott variety).

In light of the obtained antioxidant activity results and the content of the individual compounds and total phenolic compounds, a principal component analysis (PCA) and cluster analysis (CA), combined with a heat map, were conducted to determine the relationships between the investigated leaf extracts of different varieties of *Vaccinium corymbosum* L. This analysis aimed to uncover patterns and dependencies among the studied samples for potential insights into their characteristics.

**Figure 1:**
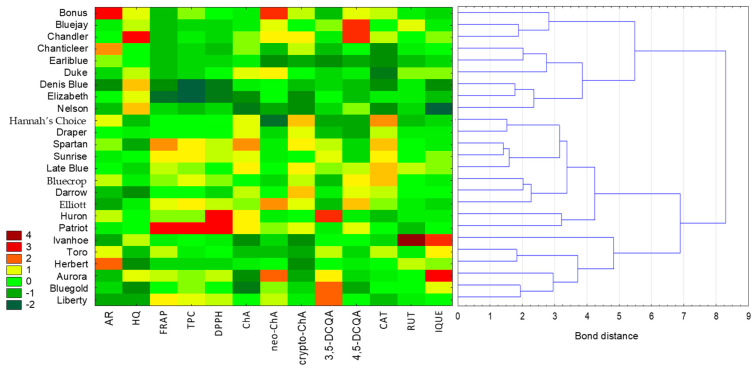
Analysis of similarity between leaf extracts of different varieties of *Vaccinium corymbosum* L. based on the content of individual identified compounds, the total content of phenolic compounds, and antioxidant activity using cluster analysis combined with a heat map. Variables were standardised. Colours on the heat map represent contents of the respective extracts, with dark red indicating high content and dark green indicating low content. (AR—arbutin; HQ—hydroquinone; ChA—chlorogenic acid; neo-ChA—neochlorogenic acid; crypto-ChA—cryptochlorogenic acid; 3,5-DCQA—3,5-dicaffeoylquinic acid; 4,5-DCQA—4,5-dicaffeoylquinic acid; CAT—catechin; RUT—rutin; IQUE—isoquercetin).

The cluster analysis was performed using the Euclidean distance as the distance measure and the Ward method as the method of combining objects (Figure 1). The analysed variables had different units, so standardisation of the values was performed. Based on the colour scale of the heat map, values of individual parameters can be compared (where the darkest red indicates the highest value of a particular compound or antioxidant activity, and the darkest green represents the lowest value). Moreover, the importance of the variables was established based on the C&RT model. The variable with the highest importance was the content of cryptochlorogenic acid (variable significance = 1), followed by the content of chlorogenic acid (0.85) and isoquercetin (0.76). The samples examined were divided into two main clusters. Extracts with the most similar values of the determined parameters are located closest to each other, but analysing the heat map reveals how significantly the analysed varieties differ from each other. For example, three varieties (Huron, Bluegold and Liberty) were characterised by the highest content of 3,5-DCQA (3,5-dicaffeoylquinic acid), while a high level of 4,5-DCQA (4,5-dicaffeoylquinic acid) was characteristic for Bluejay and Chandler. Furthermore, it can be observed how one variety (Ivanhoe) stands out in terms of rutin content compared to the other varieties. Based on the results of a chemometric analysis, it is also possible to quickly identify varieties with elevated levels of potentially toxic compounds, such Chandler with hydroquinone (HQ) content, or with unique phenolics, such as Bonus with arbutin (AR) content.

**Figure 2:**
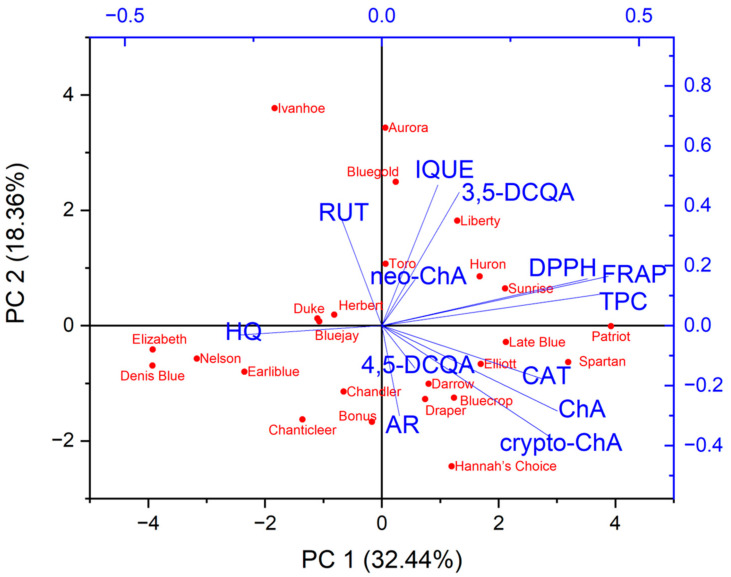
Determination of connections and relationships between leaf extracts of different varieties of *Vaccinium corymbosum* L. in relation to the analysed parameters (content of individual compounds, total content of phenolic compounds and antioxidant activity) using principal component analysis as a projection of PC1 vs. PC2. (AR—arbutin; HQ—hydroquinone; ChA—chlorogenic acid; neo-ChA—neochlorogenic acid; crypto-ChA—cryptochlorogenic acid; 3,5-DCQA—3,5-dicaffeoylquinic acid; 4,5-DCQA—4,5-dicaffeoylquinic acid; CAT—catechin; RUT—rutin; IQUE—isoquercetin).

**Figure 3:**
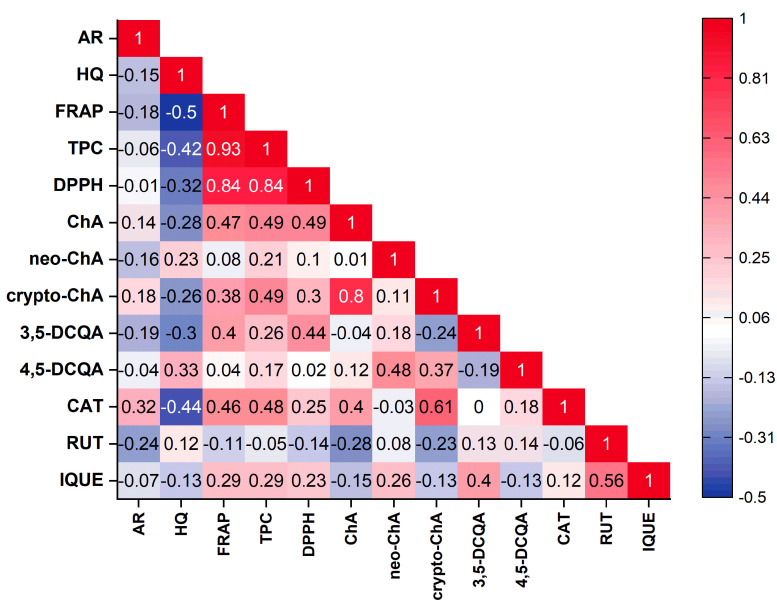
Pearson’s correlation matrix between the analysed parameters presented as a triangle heat map. (AR—arbutin; HQ—hydroquinone; ChA—chlorogenic acid; neo-ChA—neochlorogenic acid; crypto-ChA—cryptochlorogenic acid; 3,5-DCQA—3,5-dicaffeoylquinic acid; 4,5-DCQA—4,5-dicaffeoylquinic acid; CAT—catechin; RUT—rutin; IQUE—isoquercetin).

The principal component analysis (PCA) was performed to find relationships between the variables (the contents of the individual compounds, the total phenolic compounds content and antioxidant activity based on FRAP and DPPH) and the varieties of *Vaccinium corymbosum* L. Based on the Kaiser criterion, 13 variables were reduced to the four main components that explain 74.7% of the variance (PC1: 32.44%; PC2: 18.36%; PC3: 14.32%; PC4: 9.58%). Because the first two principal components carry the most information about the analysed set, the obtained results are presented as a 2D biplot (projection of PC1 and PC2) (Figure 2). The analysed varieties have not been grouped into clear groups. Similarities can be observed within specific varieties, for example, Elizabeth, Denis Blue and Nelson (left, lower part of the chart) or Hebert, Bluejay and Duke (left, upper part of the chart). Moreover, these varieties were characterised by a low content of the analysed phenolic compounds or antioxidant activity. Varieties with a higher content of a specific compound or antioxidant activity are placed on the right side of the chart. Examining the obtained biplot, correlations between the analysed variables can also be observed. However, considering that there are four principal components, direct analysis based on this plot can be misleading. Therefore, correlation results between variables were presented in the form of a triangle heat map illustrating the Pearson correlation matrix in graphical form (Figure 3). The antioxidant capacity of the tested extracts was highly correlated with the levels of the total phenolic content of DPPH vs. TPC (r = 0.84) and FRAP vs. TPC (r = 0.93), as well as the strong correlation between the FRAP and DPPH methods (r = 0.84). This confirms our previous observations [25,63]. Interestingly, only in the case of chlorogenic acid was a mean correlation observed with antioxidant activity (vs. FRAP (r = 0.47), DPPH (r = 0.49)), suggesting that this compound may be primarily responsible for shaping the antioxidant activity of blueberry leaves. However, in general, the antioxidant activity of blueberry leaf extracts depends on a synergistic interaction of multiple polyphenolic compounds. Interestingly, a negative correlation was observed between the content of hydroquinone and the antioxidant activity determined using the FRAP method (r = −0.5). Taking into account individual compounds, a correlation was observed between the content of chlorogenic acid and cryptochlorogenic acid (r = 0.8).

**Figure 4:**
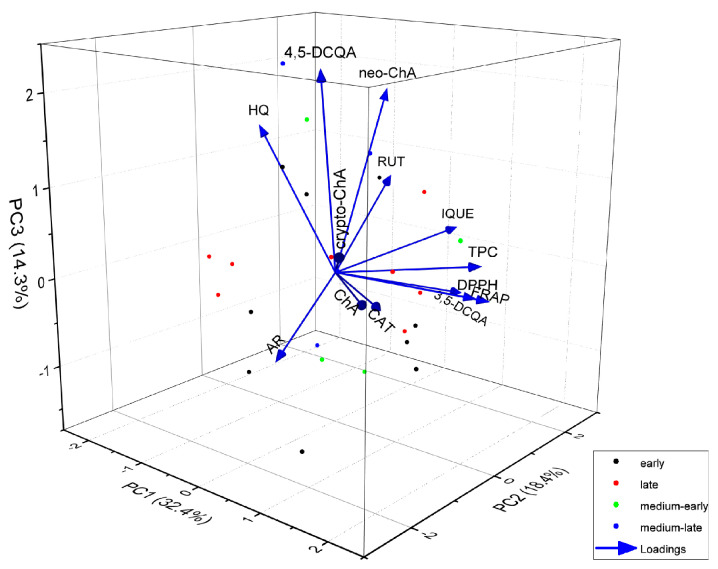
Biplot of 3D principal component analysis based on the variables (content of individual compounds, total phenolic compounds content and antioxidant activity) and analysed leaf extracts of different varieties of *Vaccinium corymbosum* L. Colours of the dots indicate the type of variety, respectively, early, late, medium-early and medium-late varieties (AR—arbutin; HQ—hydroquinone; ChA—chlorogenic acid; neo-ChA—neochlorogenic acid; crypto-ChA—cryptochlorogenic acid; 3,5-DCQA—3,5-dicaffeoylquinic acid; 4,5-DCQA—4,5-dicaffeoylquinic acid; CAT—catechin; RUT—rutin; IQUE—isoquercetin).

To explore additional relationships between the varieties, we further analysed them in relation to the harvest date and presented the findings as a 3D biplot, considering three principal components (Figure 4). As can be seen in the three-dimensional graph, taking into account the analysed variables, whether the variety is early or late does not affect the similarity of the polyphenol composition or the antioxidant activity of *Vaccinium corymbosum* L. leaves. There is also no correlation between whether a variety is early or late and the content of any specific compound. The varieties differ in genotype and, consequently, chemical composition, including the content of individual phenolic compounds, although the overall profiles are very similar. Chemometric analysis makes it possible to identify varieties rich in antioxidants, as well as varieties containing the highest content of potentially toxic compounds. Marked differences in the content of phenolic compounds and the antioxidant activity, depending on the variety, have been found in other studies [2,25,31], which confirms our results.

## 4. Conclusions

Highbush blueberry leaves contain various phytochemicals, including flavonoids and polyphenols. Analyses of 25 varieties of highbush blueberry (*V. corymbosum*.) leaf samples confirmed that the key biologically active compounds were chlorogenic acid, rutin and isoquercetin. A significant correlation was observed between the general phenolic compound content and the antioxidative activity measurement results for the analysed leaf extracts. The content of chlorogenic acid in the analysed leaf extracts ranged from 52.76 mg/g (Spartan variety) to 32.37 mg/g (Nelson variety), which represents the highest concentration among all the analysed phenolic acids. Particularly high levels of isoquercetin were found in the Aurora, Ivanhoe and Toro varieties (28.40 mg/g, 26.24 mg/g and 21.57 mg/g, respectively). An exceptionally high rutin content (*p* < 0.05) was found in the Ivanhoe variety (27.19 mg/g) as compared to the other varieties, where it ranged from 2.06 mg/g (Earliblue and Patriot varieties) to 10.55 mg/g (Bluejay variety). The Patriot variety was determined to possess the highest antioxidative activity using the FRAP method (1086.15 μmol Trolox/g d.w.) and based on its DPPH radical scavenging activity (1124.17 μmol Trolox/g d.w.). The total phenolic content (TPC) determined via spectrophotometry ranged from 48.11 mg GAE/g d.w. (Elizabeth variety) to 177.31 GAE/g d.w. (Patriot variety). The arbutin content in the leaves of all tested varieties exceeded 2%, so it can be concluded that they constitute a stable source of arbutin. Three varieties (Bonus, Chanticleer and Herbert) can be considered a potential alternative to bearberry and lingonberry leaves. The hydroquinone content in the analysed extracts was determined to be at a lower level. However, due to the relatively high arbutin levels, despite the content of valuable pro-health substances, primarily antioxidants, the leaves of the investigated highbush blueberry varieties cannot, in our opinion, be utilised directly for food products and dietary supplements. In order to use highbush blueberry leaves as a raw material for food production, selective extraction conditions should be used to obtain valuable components under mild low-temperature conditions. There is also a need to study the effect of processing, i.e., fermentation of highbush blueberry leaves, on the extraction efficiency of potentially toxic substances.

## Figures and Tables

**Table 1:** Characteristics of highbush blueberry varieties from which leaves were collected.

Variety Name	Fruit Harvest Time
Bonus, Chanticleer, Duke, Earliblue, Hannah’s Choice, Huron, Ivanhoe, Spartan, Sunrise	beginning of July
Bluejay, Draper, Patriot, Toro	end of July
Bluecrop, Chandler, Elliott, Herbert	July–August
Aurora, Bluegold, Darrow, Denis Blue, Elizabeth, Late Blue, Liberty, Nelson	August

**Table 2:** Content of phenolic acids of the tested highbush blueberry varieties (mg/g d.w.).

Variety	Chlorogenic Acid	Neochlorogenic Acid	Cryptochlorogenic Acid	3,5-Dicaffeoylquinic Acid	4,5-Dicaffeoylquinic Acid
**Aurora**	34.90 ± 1.21 ^c^	8.43 ± 0.28 ^p^	2.20 ± 0.18 ^a^	6.21 ± 0.05 ^l^	1.47 ± 0.04 ^e^
**Bluecrop**	41.65 ± 0.21 ^g^	2.94 ± 0.05 ^d,e^	3.69 ± 0.13 ^f,g,h^	2.46 ± 0.03 ^e,f^	5.72 ± 0.01 ^n^
**Bluegold**	33.39 ± 0.30 ^a,b^	5.26 ± 0.32 ^k^	2.75 ± 0.06 ^b,c^	7.63 ± 0.03 ^m^	<LOQ ^a^
**Bluejay**	40.53 ± 0.09 ^f,g^	6.41 ± 0.03 ^m^	3.17 ± 0.38 ^c,d,e^	4.89 ± 0.04 ^j^	7.79 ± 0.01 ^p^
**Bonus**	41.02 ± 0.29 ^f,g^	9.03 ± 0.21 ^r^	3.65 ± 0.10 ^e,f,g,h^	2.03 ± 0.04 ^c,d^	5.04 ± 0.02 ^m^
**Chandler**	45.88 ± 0.30 ^i,j^	6.66 ± 0.11 ^m,n^	4.00 ± 0.07 ^h,i^	3.23 ± 0.09 ^g^	7.83 ± 0.06 ^p^
**Chanticleer**	44.88 ± 0.16 ^h,i^	2.43 ± 0.13 ^d^	3.72 ± 0.04 ^f,g,h^	2.27 ± 0.01 ^d,e^	2.72 ± 0.08 ^i^
**Darrow**	46.67 ± 0.40 ^j,k^	4.58 ± 0.00 ^j,k^	4.31 ± 0.24 ^i^	3.01 ± 0.03 ^g^	4.65 ± 0.03 ^l^
**Denis Blue**	39.99 ± 0.04 ^e^	3.84 ± 0.03 ^g,h,i^	2.38 ± 0.13 ^a,b^	2.57 ± 0.04 ^f^	1.01 ± 0.01 ^b^
**Draper**	48.60 ± 0.13 ^m^	3.02 ± 0.38 ^e,f^	3.91 ± 0.04 ^h,i^	2.25 ± 0.11 ^c,d,e^	<LOQ ^a^
**Duke**	48.23 ± 0.38 ^l,m^	7.05 ± 0.08 ^n^	3.37 ± 0.06 ^d,e,f,g^	2.48 ± 0.04 ^e,f^	2.03 ± 0.07 ^g^
**Earliblue**	40.04 ± 0.20 ^e,f^	1.17 ± 0.01 ^b^	2.16 ± 0.12 ^a^	1.35 ± 0.07 ^a^	<LOQ ^a^
**Elliott**	45.86 ± 0.00 ^i,j^	7.80 ± 0.03 ^o^	3.94 ± 0.0 ^h,i^	2.00 ± 0.01 ^c^	6.20 ± 0.01 ^o^
**Elizabeth**	33.84 ± 0.24 ^b,c^	4.00 ± 0.04 ^g,h,i^	1.99 ± 0.08 ^a^	3.76 ± 0.06 ^h^	0.99 ± 0.01 ^b^
**Hannah’s Choice**	47.63 ± 0.00 ^k,l,m^	<LOQ ^a^	4.30 ± 0.03 ^i^	1.74 ± 0.04 ^b^	<LOQ ^a^
**Herbert**	40.95 ± 0.42 ^f,g^	4.33 ± 0.09 ^i,j^	2.14 ± 0.15 ^a,b^	4.43 ± 0.14 ^i^	2.00 ± 0.04 ^g^
**Huron**	49.98 ± 0.09 ^j,k,l^	3.50 ± 0.11 ^f,g^	3.03 ± 0.04 ^c,d^	8.32 ± 0.14 ^o^	1.71 ± 0.05 ^f^
**Ivanhoe**	34.21 ± 0.04 ^b,c^	2.93 ± 0.13 ^d,e^	2.08 ± 0.02 ^a^	3.90 ± 0.06 ^h^	2.46 ± 0.01 ^h^
**Late Blue**	50.52 ± 0.35 ^n^	4.16 ± 0.08 ^h,i,j^	4.12 ± 0.01 ^h,i^	5.00 ± 0.06 ^j^	4.10 ± 0.06 ^k^
**Liberty**	44.44 ± 0.21 ^h^	5.82 ± 0.08 ^i,j^	2.79 ± 0.06 ^b,c^	7.97 ± 0.02 ^n^	2.50 ± 0.01 ^h^
**Nelson**	32.37 ± 0.23 ^a^	1.73 ± 0.04 ^c^	1.99 ± 0.01 ^a^	2.57 ± 0.01 ^f^	3.87 ± 0.04 ^j^
**Patriot**	50.26 ± 0.15 ^n^	5.09 ± 0.04 ^k^	3.84 ± 0.06 ^g,h,i^	3.05 ± 0.01 ^g^	4.77 ± 0.03 ^l^
**Spartan**	52.76 ± 0.33 ^o^	3.68 ± 0.21 ^g,h^	4.14 ± 0.06 ^h,i^	5.27 ± 0.01 ^k^	1.39 ± 0.00 ^c,e^
**Sunrise**	47.79 ± 0.04 ^k,l,m^	3.67 ± 0.06 ^g,h^	3.33 ± 0.11 ^d,e,f^	5.99 ± 0.05 ^l^	1.24 ± 0.12 ^c,d^
**Toro**	36.99 ± 0.06 ^d^	2.42 ± 0.04 ^d^	2.34 ± 0.07 ^a,b^	5.26 ± 0.08 ^k^	1.20 ± 0.01 ^c^

<LOQ—below limit of quantification. Data presented as mean value ± standard deviation (SD; *n* = 3). Statistically significant differences between means (a–p) are marked by different letters in the rows (*p* ≤ 0.05).

**Table 3:** Content of flavonoids in the tested highbush blueberry varieties (mg/g d.w.).

Variety	Catechin	Rutin	Isoquercetin
**Aurora**	2.43 ± 0.28 ^d,e,f^	6.79 ± 0.18 ^i^	28.40 ± 0.17 ^n^
**Bluecrop**	3.94 ± 0.16 ^i,j^	6.09 ± 0.08 ^h^	13.57 ± 0.02 ^d,e,f^
**Bluegold**	2.48 ± 0.09 ^e,f^	6.91 ± 0.13 ^i^	19.89 ± 0.09 ^k^
**Bluejay**	2.63 ± 0.11 ^f^	10.55 ± 0.03 ^l^	14.20 ± 0.13 ^f^
**Bonus**	3.15 ± 0.08 ^g,h^	4.52 ± 0.13 ^d,e^	13.13 ± 0.08 ^d^
**Chandler**	3.24 ± 0.19 ^g,h^	7.08 ± 0.04 ^i^	17.02 ± 0.06 ^h,i^
**Chanticleer**	1.40 ± 0.12 ^a^	4.04 ± 0.06 ^b,c,d^	15.40 ± 0.08 ^g^
**Darrow**	3.21 ± 0.06 ^g,h^	5.51 ± 0.01 ^g,h^	15.53 ± 0.32 ^g^
**Denis Blue**	1.41 ± 0.05 ^a,b^	3.86 ± 0.09 ^b,c^	8.75 ± 0.07 ^b^
**Draper**	3.19 ± 0.00 ^g,h^	4.71 ± 0.07 ^e,f^	14.20 ± 0.04 ^f^
**Duke**	1.35 ± 0.03 ^a^	8.07 ± 0.06 ^j^	17.25 ± 0.00 ^i,j^
**Earliblue**	1.53 ± 0.00 ^a,b,c^	2.06 ± 0.05 ^a^	13.18 ± 0.02 ^d,e^
**Elliott**	2.92 ± 0.32 ^f,g^	4.49 ± 0.02 ^d^	12.06 ± 0.05 ^c^
**Elizabeth**	2.40 ± 0.05 ^d,e,f^	4.16 ± 0.04 ^b,c,d,e^	11.43 ± 0.07 ^c^
**Hannah’s Choice**	4.15 ± 0.06 ^j^	2.38 ± 0.05 ^a^	13.00 ± 0.05 ^d^
**Herbert**	2.90 ± 0.18 ^f,g^	8.71 ± 0.12 ^k^	17.07 ± 0.05 ^h,i^
**Huron**	1.95 ± 0.05 ^c,d^	4.08 ± 0.01 ^b,c,d^	13.80 ± 0.22 ^e,f^
**Ivanhoe**	2.06 ± 0.15 ^d,e^	27.19 ± 0.59 ^m^	26.24 ± 0.02 ^m^
**Late Blue**	3.91 ± 0.01 ^i,j^	9.06 ± 0.08 ^k^	17.85 ± 0.02 ^j^
**Liberty**	1.91 ± 0.03 ^b,c,d^	4.67 ± 0.03 ^e,f^	15.01 ± 0.00 ^g^
**Nelson**	1.46 ± 0.16 ^a,b,c^	3.61 ± 0.04 ^b^	5.13 ± 0.04 ^a^
**Patriot**	2.88 ± 0.13 ^f,g^	2.06 ± 0.09 ^a^	16.51 ± 0.29 ^h^
**Spartan**	3.95 ± 0.04 ^i,j^	5.14 ± 0.01 ^f,g^	14.00 ± 0.02 ^f^
**Sunrise**	3.60 ± 0.14 ^h,i^	5.25 ± 0.13 ^f,g^	17.34 ± 0.09 ^i,j^
**Toro**	3.19 ± 0.02 ^g,h^	4.27 ± 0.01 ^c,d,e^	21.57 ± 0.55 ^l^

<LOQ—limit of quantification. Data presented as mean value ± standard deviation (SD; *n* = 3). Statistically significant differences between means (a–n) are marked by different letters in the rows (*p* ≤ 0.05).

**Table 4:** Content of arbutin and hydroquinone in the tested highbush blueberry varieties.

Variety	Arbutin		Hydroquinone	
	mg/g d.w.	%	mg/g d.w.	%
**Aurora**	24.30 ± 1.87 ^a,b,c^	2.43	0.62 ± 0.01 ^k,l.m^	0.06
**Bluecrop**	34.21 ± 1.56 ^g,h^	3.42	0.42 ± 0.03 ^d,e,f,g,h^	0.04
**Bluegold**	25.41 ± 0.84 ^b,c,d^	2.54	0.24 ± 0.02 ^a^	0.02
**Bluejoy**	27.01 ± 1.39 ^c,d,e,f^	2.70	0.59 ± 0.06 ^i,j,k^	0.06
**Bonus**	44.24 ± 3.84 ^j^	4.42	0.61 ± 0.04 ^j,k,l^	0.06
**Chandler**	30.18 ± 1.61 ^d,e,f,g^	3.02	0.88 ± 0.02 ^n^	0.09
**Chanticleer**	40.01 ± 1.23 ^i,j^	4.00	0.52 ± 0.02 ^h,i,j,k^	0.05
**Darrow**	24.05 ± 1.96 ^a,b,c^	2.40	0.26 ± 0.02 ^a,b^	0.03
**Denis Blue**	19.94 ± 1.63 ^a^	1.99	0.73 ± 0.05 ^m^	0.07
**Draper**	30.27 ± 1.07 ^e,f,g^	3.03	0.40 ± 0.06 ^c,d,e,f^	0.04
**Duke**	24.97 ± 1.39 ^b,c^	2.50	0.59 ± 0.05 ^i,j,k^	0.06
**Earliblue**	31.51 ± 1.15 ^f,g,h^	3.15	0.51 ± 0.02 ^g,h,i,j^	0.05
**Elliott**	21.16 ± 1.23 ^a,b^	2.12	0.41 ± 0.01 ^c,d,e,f,g^	0.04
**Elizabeth**	30.38 ± 1.71 ^e,f,g^	3.04	0.62 ± 0.04 ^k,l,m^	0.06
**Hannah’s Choise**	35.69 ± 1.63 ^h,i^	3.57	0.31 ± 0.03 ^a,b,c^	0.03
**Herbert**	41.64 ± 1.11 ^j^	4.16	0.26 ± 0.01 ^a,b^	0.03
**Huron**	32.70 ± 0.75 ^g,h^	3.27	0.52 ± 0.02 ^g,h,i,j^	0.05
**Ivanhoe**	21.14 ± 0.28 ^a,b^	2.11	0.60 ± 0.03 ^i,j,k^	0.06
**Late Blue**	30.14 ± 1.05 ^d,e,f,g^	3.01	0.36 ± 0.01 ^b,c,d,e^	0.04
**Liberty**	21.15 ± 0.97 ^a,b^	2.11	0.27 ± 0.02 ^a,b^	0.03
**Nelson**	23.31 ± 1.06 ^a,b,c^	2.33	0.71 ± 0.03 ^l,m^	0.07
**Patriot**	26.52 ± 1.32 ^c,d,e^	2.65	0.50 ± 0.03 ^f,g,h,i^	0.05
**Spartan**	31.12 ± 0.48 ^e,f,g,h^	3.11	0.28 ± 0.01 ^a,b^	0.03
**Sunrise**	26.65 ± 1.44 ^c,d,e^	2.66	0.45 ± 0.01 ^e,f,g,h^	0.04
**Toro**	34.51 ± 1.89 ^g,h^	3.45	0.34 ± 0.02 ^a,b,c,d^	0.03

Data as mean value ± standard deviation (SD; *n* = 3). Statistically significant differences between means (a–n) are marked by different letters in the rows (*p* ≤ 0.05).

**Table 5:** Antioxidant activity of the tested highbush blueberry varieties.

Variety	FRAP μmol Trolox/g d.w.	TPC mg GAE/g d.w.	DPPH μmol Trolox/g d.w.
**Aurora**	793.85 ± 10.88 ^j,k^	121.44 ± 2.04 ^f,g,h,i,j^	792.78 ± 4.05 ^j,k^
**Bluecrop**	758.08 ± 16.86 ^i,j^	129.01 ± 1.02 ^g,h,i,j^	751.21 ± 16.22 ^h,i,j^
**Bluegold**	718.08 ± 14.69 ^h,i^	118.74 ± 5.35 ^e,f,g,h,i^	730.43 ± 5.12 ^g,h^
**Bluejay**	549.23 ± 1.09 ^c,d,e^	89.73 ± 1.53 ^b,c^	573.47 ± 24.33 ^b^
**Bonus**	552.69 ± 15.77 ^c,d^	114.95 ± 12.23 ^d,e,f,g^	665.21 ± 24.33 ^e,f^
**Chandler**	537.31 ± 2.72 ^c,d^	94.77 ± 1.05 ^b,c,d^	579.21 ± 4.05 ^b^
**Chanticleer**	521.15 ± 13.62 ^c^	87.93 ± 1.10 ^b,c^	556.98 ± 15.24 ^b^
**Darrow**	661.54 ± 17.41 ^f,g^	101.62 ± 1.15 ^b,c,d,e,f^	606.44 ± 4.05 ^b,c,d^
**Denis Blue**	433.08 ± 8.70 ^b^	49.19 ± 0.76 ^a^	400.74 ± 19.26 ^a^
**Draper**	685. 00 ± 2.72 ^g,h^	114.05 ± 1.78 ^d,e,f,g^	681.69 ± 3.04 ^e,f,g^
**Duke**	541.15 ± 20.13 ^c,d,e^	90.45 ± 12.74 ^b,c^	661.62 ± 1.01 ^d,e,f^
**Earliblue**	513.85 ± 4.35 ^c^	87.39 ± 9.94 ^b,c^	599.99 ± 23.31 ^b,c^
**Elliott**	840.77 ± 10.88 ^l,m^	136.22 ± 5.13 ^h,i,j^	798.65 ± 2.21 ^j,k^
**Elizabeth**	350.77 ± 6.53 ^a^	48.11 ± 0.76 ^a^	403.65 ± 8.83 ^a^
**Hannah’s Choice**	676.54 ± 4.92 ^g,h^	107.75 ± 4.59 ^c,d,e,f^	715.93 ± 24.29 ^f,g,h^
**Herbert**	573.85 ± 11.97 ^d,e^	88.11 ± 0.25 ^b,c^	608.96 ± 16.56 ^b,c,d^
**Huron**	763.08 ± 8.71 ^j^	116.04 ± 1.00 ^e,f,g,h^	1144.47 ± 12.14 ^l^
**Ivanhoe**	628.85 ± 7.07 ^f^	106.49 ± 7.39 ^c,d,e,f^	642.52 ± 4.42 ^c,d,e^
**Late Blue**	809.62 ± 13.60 ^k,l^	118.21 ± 2.55 ^e,f,g,h,i^	736.98 ± 3.31 ^g,h,i^
**Liberty**	853.85 ± 10.88 ^m,n^	129.91 ± 0.76 ^g,h,i,j^	790.84 ± 8.83 ^i,j,k^
**Nelson**	583.08 ± 2.18 ^e^	83.60 ± 3.57 ^b^	574.61 ± 7.73 ^b^
**Patriot**	1086.15 ± 8.70 ^p^	177.31 ± 3.57 ^k^	1124.17 ± 3.31 ^l^
**Spartan**	938.46 ± 1.09 ^o^	141.98 ± 2.57 ^j^	809.58 ± 6.62 ^k^
**Sunrise**	885.77 ± 7.07 ^n^	138.56 ± 5.35 ^i,j^	814.26 ± 4.42 ^k^
**Toro**	783.46 ± 5.98 ^j,k^	98.23 ± 4.84 ^b,c,d,e^	711.22 ± 24.29 ^f,g,h^

Data presented as mean value ± standard deviation (SD; *n* = 3). Statistically significant differences between means (a–p) are marked by different letters in the rows (*p* ≤ 0.05).

## Data Availability

The data presented in this study are available on request from the corresponding author.
